# Antihypertensive drugs use and the risk of prostate cancer: a meta-analysis of 21 observational studies

**DOI:** 10.1186/s12894-018-0318-7

**Published:** 2018-03-07

**Authors:** Liang Cao, Sha Zhang, Cheng-ming Jia, Wei He, Lei-tao Wu, Ying-qi Li, Wen Wang, Zhe Li, Jing Ma

**Affiliations:** 1 Department of Traditional Chinese Medicine, Xijing Hospital, Fourth Military Medical University, Xi’an, 710032 People’s Republic of China; 2 Shanxi University of Chinese Medicine, Xian yang, 712046 People’s Republic of China

**Keywords:** Antihypertensive drugs, Prostate cancer, Meta-analysis

## Abstract

**Background:**

Due to the lack of strong evidence to identify the relationship between antihypertensive drugs use and the risk of prostate cancer, it was needed to do a systematic review to go into the subject.

**Methods:**

We systematically searched PubMed, Web of Science and Embase to identify studies published, through May 2015. Two evaluators independently reviewed and selected articles involving the subject. We used the Newcastle-Ottawa Scale (NOS) to assess the quality of the studies. All extracted results to evaluate the relationship between antihypertensive drugs usage and prostate cancer risk were pool-analysed using Stata 12.0 software.

**Results:**

A total of 12 cohort and 9 case-control studies were ultimately included in our review. Most of the studies were evaluated to be of high quality. There was no significant relationship between angiotensin converting enzyme inhibitors (ACEI) usage and the risk of prostate cancer (RR 1.07, 95% CI 0.96–1.20), according to the total pool-analysed. Use of angiotensin receptor blocker (ARB) was not associated with the risk of prostate cancer (RR 1.09, 95% CI 0.97–1.21), while use of CCB may well increase prostate cancer risk based on the total pool-analysed (RR 1.08, 95% CI 1–1.16). Moreover, subgroup analysis suggested that use of CCB clearly increased prostate cancer risk (RR 1.10, 95% CI 1.04–1.16) in terms of case-control studies. There was also no significant relationship between use of diuretic (RR 1.09, 95% CI 0.95–1.25) or antiadrenergic agents (RR 1.22, 95% CI 0.76–1.96) and prostate cancer risk.

**Conclusions:**

There is no significant relationship between the use of antihypertensive drugs (ACEI, ARB, beta-blockers and diuretics) and prostate cancer risk, but CCB may well increase prostate cancer risk, according to existing observational studies.

**Electronic supplementary material:**

The online version of this article (10.1186/s12894-018-0318-7) contains supplementary material, which is available to authorized users.

## Background

The prevalence of hypertension is consistently high in older adults and regarded as a vital risk factor for cardiovascular diseases, congestive heart failure, and coronary heart disease [1, 2]. Antihypertensive drugs including angiotensin-converting enzyme inhibitors (ACEI), angiotensin II receptor blockers (ARB), calcium-channel blockers (CCB), alpha- and beta-blockers and diuretics, were mainly used for the control of blood pressure in patients with hypertension to prevent relevant cardiovascular diseases [3]. Beneficial therapeutic effects of these drugs, on blood pressure control, have been well established in previous literature [4].

However, antihypertensive drugs and cancer risk have long been raised as concerns [5]. It was first reported that Rauwolfia derivatives increased the risk of breast cancer [6]. After that, several classes of antihypertensive agents appeared to elevate cancer risk, but the relationship between antihypertensive drug usage and increased cancer risk could not be confirmed in numerous studies due to their short follow-up and the cancer risk from hypertension itself [7]. A retrospective cohort study showed that the use of ACE inhibitors has an association with a clearly decreased risk of overall cancer [8]. Meanwhile, a meta-analysis demonstrated that there was no association between the use of ACE inhibitors or angiotensin-receptor blockers and the overall risk of cancer [9]. A large-scale, population-based cohort study proposed that there was no substantial association between the use of calcium channel blockers (CCB) and the incidence rate of cancer or cancer mortality [10]. In a study by Hershel et al. study, there was small positive association between CCB usage and risk of cancer [11].

The incidence of prostate cancer is increasing, and it is the main cause of cancer death in males in the Western countries [12]. Studies on the association between antihypertensive drug usage and prostate cancer risk remain controversial. In vitro studies, CCB enhanced apoptosis of prostate cancer cells and might have a protective effect on prostate cancer [13]. Debes et al. found that CCB significantly decreased the risk of prostate cancer, and their results varied by family history of prostate cancer [14]. However, some case-control studies did not find the this relationship [15]. For instance, a study with 1,165,781 patients did not support the association between the long-term use of CCB and prostate cancer risk [16]. Some case-control studies showed significantly increased risk between ACE inhibitor usage and prostate cancer [15, 17], while a previous meta-analysis showed that ACE inhibitors or angiotensin-receptor blockers did not affect the occurrence of cancer [18]. In a study by Perron et al., beta-blockers were associated with a reduction in prostate cancer risk, while another study by Kemppainen et al. tended to show an increased risk of prostate cancer in patients treated by beta-blockers [15, 19]. One study reported the relationship between alpha- blockers or diuretic usage and prostate cancer risk and their results were also controversial [20].

Due to there being the long-term follow-up in the observational studies, we performed a systematic review of observational studies to confirm whether the use of antihypertensive drugs was able to result in the prostate cancer in the human body.

## Methods

Our review was conducted in accordance with PRISMA (Preferred Reporting Items for Systematic Reviews and Meta-Analyses) and MOOSE (Meta-Analysis of Observational Studies in Epidemiology) guidelines.

### Eligibility criteria

Studies were included if they met the following points: (1) Studies were cohort or case-control studies; and (2) the relationship between the use of one or more types of antihypertensive drugs and prostate cancer was reported in the study.

### Search strategy

We systematically searched PubMed, Web of Science and Embase to identify studies published through May 2015. The search terms were composed of the following: “beta blockers”, “angiotensin converting enzyme inhibitors”, “angiotensin receptor blockers”, “calcium channel blockers” “alpha blockers” “antihypertensive drugs” and “prostate cancer”. The details of the search methods are summarized in the Additional file 1. We also screened the bibliographies of relevant articles to find additional articles that met the included standard. A language limitation was not set during the search process. We did not consider animal studies when we reviewed the records.

### Study selection and data extraction

Two authors independently evaluated the studies retrieved from the databases to select the studies that met the inclusion criteria. Disagreements between the two reviewers were resolved by discussion or in consultation with an arbitrator. The following information was extracted from the included articles by the two authors independently: study design, geographic region, type of medication, sex, age range, follow-up time, adjusted factors in each study (Table1). The RR (relative risk), HR (hazard ratio), OR (odds ratio), SIR (standardized incidence ratio) along with their corresponding 95%CI (confidence interval), all of which indicated the relationship between antihypertensive drugs and prostate cancer were abstracted. If there was missing information in the article, we contacted the authors via e-mail or telephone. We used data from a 2 × 2 table to recalculate crude estimates when the outcome measures were unsuitable for meta-analysis and we failed to gain the data from the authors.

### Methodological quality assessment

The quality of the observational studies was independently evaluated by two authors based on the Newcastle-Ottawa Assessment Scale(NOS) on the website (http://www.ohri.ca/programs/clinical_epidemiology/oxford.asp). The scale provided eight items consisting of three subscales: selection of subjects (four items), comparability of subjects (one item) and assessment of outcome/exposure (three items). The highest scores were nine for the eight items because there were two scores in the comparability of subjects. A study with greater than or equal to seven scores was considered to be of high methodological quality.

### Data synthesis and analysis

Extracted RRs, HRs, ORs and SIRs and their 95% CIs that were adjusted for most confounders were pooled to compute the RR between antihypertensive drugs and prostate cancer risk [21]. The four measures of association above were expected to yield similar estimates of RR, due to the incidence of prostate cancer being generally low [22]. For a single study that reported more than one type of cancer, only the data on the risk between prostate cancer and antihypertensive drugs were extracted and then pooled. If there were studies involving multi-drug treatment and we were not familiar with the data that reported which specific drug was associated with the incidence of prostate cancer in these studies, the data of the study would not be extracted to conduct a meta-analysis. In the included studies, the data that reported the risk between a single antihypertensive drugs and the occurrence of prostate cancer will be pooled for analysis based on the single drug category respectively. The meta-analysis was performed with Stata 12.0. Since the clinical and methodological heterogeneity were known, we used a random-effects model to calculate pooled RRs and their 95%CIs. Subgroup analyses were performed according to whether they corresponded to case-control or cohort studies. Between-study heterogeneity was assessed by using Cochran’s Q statistic (significance level at *p* < 0.1) and by estimating I^2^. If I^2^ was more than 40%, there was significant heterogeneity among studies [23].

## Results

### Characteristic of included studies

**Fig. 1:**
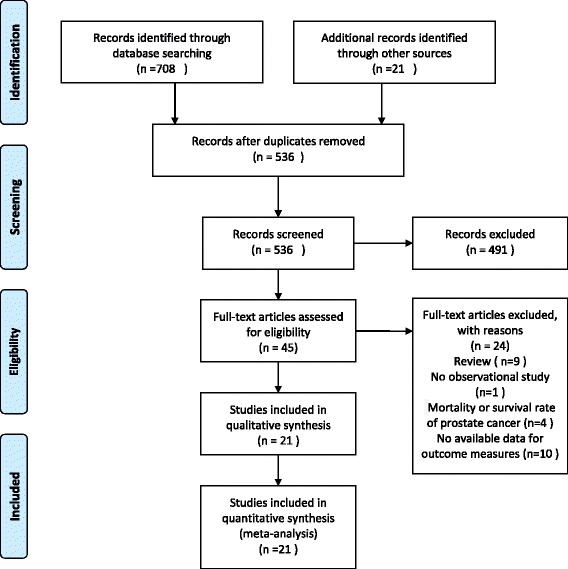
PRISMA flow diagram

Our search strategy yielded 729 records, A total of 193 and 491 records were excluded due to duplicated records and irrelevant subjects respectively. A total of 45 full-text articles were assessed and 21 studies that met our criteria were ultimately included (Fig. 1). These studies included 12 cohort studies [10, 20, 24–33], 4 nested case-control studies [11, 16, 34, 35] and 5 case-control studies [15, 17, 19, 36, 37]. The follow-up time in most cohort studies was more than 5 years. There were 11 studies that involved males only [10, 15, 19, 20, 24, 26, 28, 31, 35, 36, 38]. It was reported that the main outcomes of the included studies were adjusted for most of the confounding factors, and this information was missing in two studies (Tables 1 and 2) [10, 32].

**Table 1:** Characteristics of cohort studies included in the meta-analysis

Studies	Types of studies	Population and slection of cases	NO. of participants	Type of medication (reference group)	Duration of follow-up,yr	Sex (%)	Mean age (range), yr	Adjustment
Pai, P. Y.et al. 2015 [20]	cohort study	Male patients with hypertension or without hypertension selected from CCHIA-NHI database	80,299	Diuretics, Alpha-blockersBeta-blockers, ARBsCCBs, ACEIOthers (no use of antihypertensive drugs)	9	Male (100)	69.28 VS 69.31(50-)	Age, urbanization level, income, comorbidities
Rao, G. A. et al. 2013 [24]	cohort study	Males patients receiving drug treatment from VA of U.S.A.	543,824	ARBs (no use of ARBs)	8	Male (100)	63.6 VS 63.6	All 54 variables that was used to compute propensity to receive treatment
Bhaskaran, K. et al. 2012 [25]	cohort study	Hypertensive patients receiving drug treatment from General Practice Research Database (GPRD) of U.K.	377,649	ARBs (no use of ARBs)	>5	M (52) F (48)	64 (18–103)	Age, sex, BMI, smoking, alcohol, diabetes (with or without metformin/insulin use), hypertension, heart failure, statin use, index of multiple deprivation score, calendar year.
Rodriguez, C. 2009 [26]	cohort study	Males patients receiving drug treatment from the CPS-II Nutrition Cohort of U.S.A	3031	CCBs, Beta-blockers, ACEIs, diuretics, and other anti-hypertensives (no use of anti-hypertensive drugs)	8	Males (100)	NA	Age at interview, race, education, BMI in 1997, family history of prostate cancer, history of diabetes, history of PSA screening,history of heart disease or bypass surgery, and use of cholesterol-lowering drugs
van der Knaap, R. et al. 2008 [27]	cohort study	Eligible individuals from the Rotterdam Study started with a baseline interview between July 1989 and July 1993.	7983	ACEI and/or angiotensin II type 1 receptor antagonist (no use of the drugs)	9.6	M (38.7) F (61.3)	70.4(50-)	Age, BMI, use of salicylates, diabetes mellitus, hypertension, and myocardial infarction.
Harris, A. M. et al. 2007 [28]	cohort study	Male patients receiving drug treatment seen at Lexington Veterans Affairs (VA) Hospital	27,138	α1-blockers (no use of α1-blockers)	>5	Male (100)	68 (50–89) VS 72 (46–99)	Unadjusted
Debes, J. D. et al. 2004 [29]	cohort study	Males from subgroup of Olmsted County Study of Urinary Symptoms and Health Status	2115	CCBs (no use of CCBs)	10	Male (100)	NA(40–79)	Age and family history of prostate cancer
Friis, S. et al. 2001 [30]	cohort study	Persons receiving drug treatment from Pharmacoepidemiological Prescription Research Database of North Jutland County, Denmark,	17,897	ACEI (no use of ACEI)	8	Male (50) Female (50)	62(NA)	Adjustment for age, gender, and duration of follow-up
Fitzpatrick, A. L. 2001 [31]	cohort study	Individuals receiving drug treatment from chrot of the Cardiovascular Health Study (CHS) of USA	2442	CCBsACEIβ-blockersDiureticVasodilator (no use of antihypertensive drugs)	5.6	Male (100)	NA (65-)	Adjusted for age, race (black), and body mass index (BMI)
Sorensen, H. T. 2000 [10]	cohort study	Individuals taking CCBs from Pharmaco-Epidemiological Prescription Database of the County of North Jutland, Denmark	23, 167	CCBs (compared with the number expected, based on population rates from the Danish Cancer Registry)	3.2	Male (100)	63.4	NA
Olsen, J. H. 1997 [32]	cohort study	Individuals receiving treatment of CCBs from the County of North Jutland	17,911	CCBs (compared with the number expected, based on population rates from the Danish Cancer Registry)	1.8 years	Male (49), Female (51)	NA	NA
Pahor, M. 1996 [33]	cohort study	Individuals aged 65 years or older living in East Boston, Massachusetts, and in the counties of Iowa and Washington in the state of Iowa from epidemiologic studies of the elderly (EPESE) in U.S.	5052	CCBs (no use of CCBs)	3.6	Male (35.7), Female (64.3)	MA (65-)	Adjusted for age, sex, ethnic origin, heart failure, number of hospital admissions, cigarette smoking, and alcohol intake.

*CCB* calcium-channel blockers, *ACEI* angiotensin-converting enzyme inhibitors, *ARB* angiotensin II receptor blockers, *NA* not available

**Table 2:** Characteristics of case-control studies included in the meta-analysis

Studies	Types of studies	Case selection	NO. of participants	Collection of medication data (period)	Age of cases, yr., mean (range)	Sex of cases, %	Type of drugs (reference group)	Adjustment
Hallas, J. 2012 [17]	Case control	Review of data from the Danish Cancer Registry (DCR), the Danish National Registry of Patients (DNRP),the Prescription Database of the DanishMedicines Agency and the Danish Person Registry (2000–2005).	149, 417	Review of electronic medical records (1995 until cancer diagnosis)	69.4	Male (47.7), Female (52.3)	Use of ARBs or ACEI (never-use of the durgs)	(1)chronic obstructive pulmonary disease (COPD) as a crude marker of heavy smoking; (2) inflammatory bowel disease; (3) a modified Charlson Index that contains 19 categories of comorbidity and each category has an associated weight based on the adjusted risk of 1 year mortality; (4) non-steroidal antiinflammatory drugs (NSAIDs) or hig dose aspirin, oestrogen hormone therapy, oral contraceptives, finasteride or statins.
Azoulay, L. 2012 [39]	Nested case-control	Review of data from General Practice Research Database (GPRD)in U.K. (1995–2010)	1,165,781	Review of computerized medical records (1995 until cancer diagnosis)	72.4	Male (52.7), Female (47.3)	use of ARBs or ACEIs or CCBs or alpha-blockers(use of Diuretics and/or beta-blockers)	Excessive alcohol use, body mass index, smoking, diabetes, previous cancer, and ever of aspirin, statins, and NSAIDs. In addition,cholecystectomy, inflammatory bowel disease and history of polyps for colorectal cancer; benign prostatic hyperplasia, 5-alpha reductase inhibitors, and number of PSA tests for prostate cancer; oophorectomy, use of hormone replacement therapy, and prior use of oral contraceptives for breast cancer.
Kemppainen, K. J. 2011 [15]	Case control	Review of data from the Finnish Cancer Registry (1995–2002)	25,029	Review of the prescription database of the Social Insurance Institution of Finland (1995 until cancer diagnosis)	NA	Males (100)	use of ARBs or ACEIs or CCBs or alpha-blockers or beta-blockers or diuretics (Nonusers of any antihypertensive medication)	Adjusted for age, place of residence, and use of cholesterol-lowering drugs, antidiabetic drugs, finasteride, or alpha-blockers.
Assimes, T. L. 2008 [34]	Nested case-control	Review of computerized database files of Saskatchewan Health (1980–2003)	11,697	Review of the linkable databases including the world’s oldest electronic prescription database (1978 until cancer diagnosis)	71.8	Male (53.2) Female (46.8)	Use of β-blockers or CCBs or RAS inhibitors and never use of thiazide diuretics (use of thiazide diuretics and never use of β-blockers or CCBs or RAS inhibitors)	Adjusted for age, all measured comorbid conditions, and exposure to all other classes of antihypertensive not of interest except for potassium sparing diuretics.
Ronquist, G. 2004 [35]	Nested case-control	Review of the General Practice Research Database (GPRD) in U.K. (1995–1999)	243,331	Review of computerized medical records (1995 untilcancer diagnosis)	50–79	Males (100)	Use of diuretics, beta-blockers, ACE-inhibitors, CCBs, alpha-blockers and other antihypertensives (no use)	Adjusted for age, calendar year, prostatism and and other variables.
Perron, L. 2004 [19]	case-control	Review of the source population in Quebec cancer registry (1993–1995)	13,326	Review of computerized medical records (1981 untilcancer diagnosis)	75.7	Males(100)	Use of CCBs or ACEIs or beta-blockers or thiazidic diuretics and similars or others inlclusing vasodilatators and centrally acting adrenocep-tor antagonists. (no use)	Adjusted for age, recent medical contacts, and Aspirin use
Vezina, R. M. 1998 [36]	case-control	Monthly contact with the tumor registrar and review of Massachusetts Cancer Registry for males less than 70 years of age diagnosed with prostate cancers in Massachusetts (1992–1995)	2617	Telephone interview (lifetime until cancer diagnosis)	64	Males(100)	Use of CCBs or beta-blockers or ACEIs or Thiazides or others (no use)	Age; race; level of education; family history of prostate cancer; dietary fat intake; BMI; alcohol, tobacco, and coffee use; urologic symptoms; and physician visits 2 years previously.
Rosenberg, L. 1998 [37]	case-control	Interviewed patients aged 40 to 69 years in Boston, Mass, New York, NY, Philadelphia, Pa,and Baltimore, Md (1976–1996)	16,005	Interview with standard questionnaires by trained nurse (lifetime until cancer diagnosis)	56(40–69)	Males (41) Females (59)	Use of CCBs or beta-blockers or ACEIs (no use)	Age, BMI, interview year, annual visits to a physician 2 yr. before admission, smoking amount(pack year) for all cancers, and other additional risk factors for regressions for each cancer site)
Jick, H.1997 [11]	Nested case-control	Review of all hypertensive patients on the General Practice Research Database (GPRD) who were current users of beta-blockers only, ACEIs only, or CCBs only (with or without diuretics) and who had a first-time diagnosis of any cancer recorded in 1995.	2196	Review of computerized medical records (1987 until cancer diagnosis)	71.6 (NA)	Males (49.6) Females (50.4)	Use of CCBs (use of beta-blockers)	Smoking, BMI, change of medication, duration of hypertension, and diuretic use

*CCB* calcium-channel blockers, *ACEI* angiotensin-converting enzyme inhibitors, *ARB* angiotensin II receptor blockers, *NA* not available

### Quality of included studies

The results of the quality assessment for the included studies are summarized in Tables 3 and 4. Quality scores for cohort studies ranged between 5 and 9, and those for case-control studies ranged between 7 and 9. Five studies showed that their outcomes of interest were not present at the start of the study. Thirteen studies gained two scores in the section of comparability due to their well the control of confounding factors [15, 17, 24–27, 31, 33, 34–37, 39]. There was only one study whose ascertainment of exposure was deruved from self-report [26]. The duration of follow-up in two studies was less than 5 years [10, 32]. The non-response rate was low in the included cohort studies but the scores for this item were lacking in the case-control studies.

**Table 3:** Assessment of the methodologic quality of the cohort studies included in meta-analysis

Studies	Slection				Comparability		Outcome			Total scores
	1	2	3	4	1	2	1	2	3	
Pai, P. Y.et al. 2015 [20]	+	+	+	+	+		+	+	+	8
Rao, G. A. et al. 2013 [24]	+	+	+	+	+	+	+	+	+	9
Bhaskaran, K. et al. 2012 [25]	+	+	+	+	+	+	+	+	+	9
Rodriguez, C. 2009 [26]	+	+	+	+	+	+		+	+	8
van der Knaap, R. et al. 2008 [27]	+	+	+	+	+	+	+	+	+	9
Harris, A. M. et al. 2007 [28]	+	+	+				+	+		5
Debes, J. D. et al. 2004 [29]	+	+	+	+	+		+	+	+	8
Friis, S. et al. 2001 [30]	+	+	+		+		+	+	+	7
Fitzpatrick, A. L. 2001 [31]	+	+	+	+	+	+	+	+	+	9
Sorensen, H. T. 2000 [10]	+	+	+				+		+	5
Olsen, J. H. 1997 [32]	+	+	+				+		+	5
Pahor, M. 1996 [33]	+	+	+	+	+	+	+	+	+	9

+: the article gain 1 score in the item

**Table 4:** Assessment of the methodologic quality of the case-control studies included in meta-analysis

Studies	Slection				Comparability		Exposure			Total scores
	1	2	3	4	1	2	1	2	3	
Hallas, J. 2012 [17]	+	+	+	+	+	+	+	+	+	9
Azoulay, L. 2012 [39]	+	+	+	+	+	+	+	+		8
Kemppainen, K. J. 2011 [15]	+	+	+		+	+	+	+		7
Assimes, T. L. 2008 [34]	+	+	+	+	+	+	+	+		8
Ronquist, G. 2004 [35]	+	+	+	+	+	+	+	+		8
Perron, L. 2004 [19]	+	+	+	+	+		+	+		7
Vezina, R. M. 1998 [36]	+	+	+	+	+	+	+	+		8
Rosenberg, L. 1998 [37]	+	+	+	+	+	+	+	+	+	9
Jick, H. 1997 [11]	+	+	+	+	+		+	+		7

+: the article gain 1 score in the item

### ACEI and prostate cancer risk

**Fig. 2:**
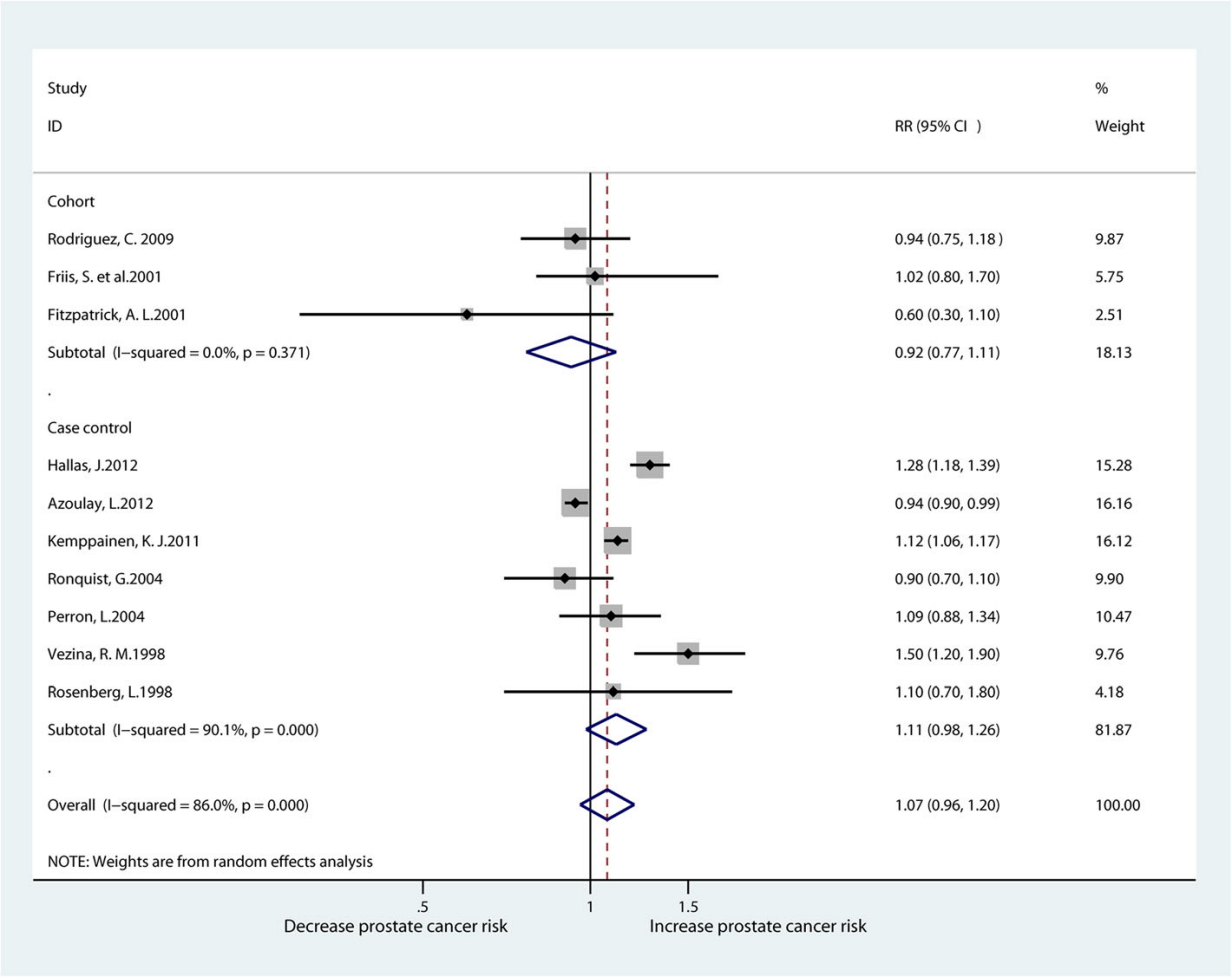
Forest plot for ACEI use and prostate cancer risk (RR relative risk, CI confidence interval)

There were ten studies that reported the relationship between the use of ACE inhibitors and the risk of prostate cancer [15–17, 19, 26, 30, 31, 35–37]. We found no significant association between ACE inhibitor usage and the risk of prostate cancer in the meta-analysis of the ten studies (RR1.07, 95% CI0.96–1.20). However, obvious clear heterogeneity existed among these studies (I^2^ = 86%). Subgroup analysis also showed no significant relationship between the use of ACE inhibitor and the risk of prostate cancer according to the poolanalysis of cohort studies (RR0.92, 95% CI0.77–1.11) and case-control studies (RR1.11, 95% CI0.98–1.26) (Fig. 2).

### ARB and prostate cancer risk

**Fig. 3:**
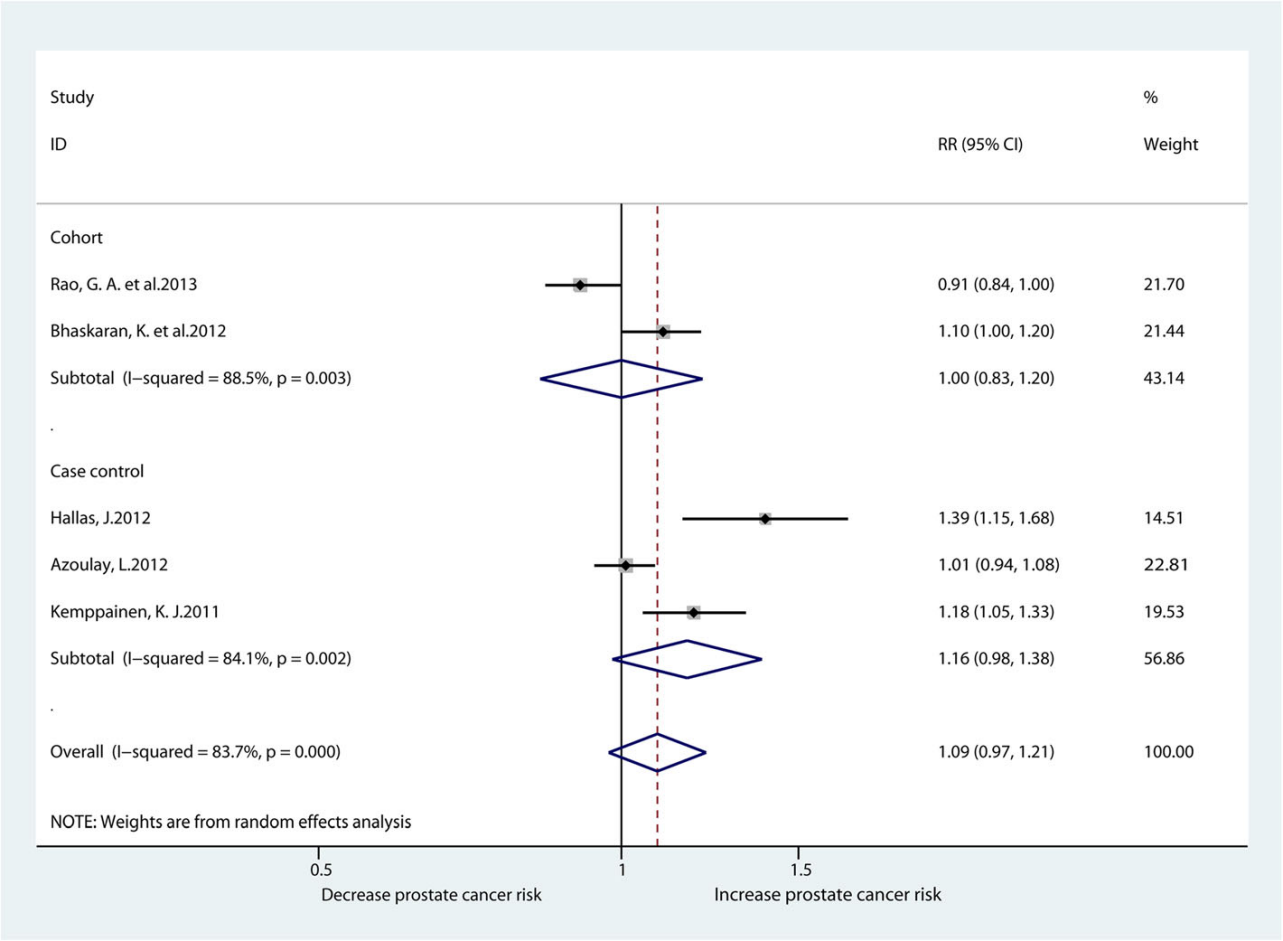
Forest plot for ARB use and prostate cancer risk (RR relative risk, CI confidence interval)

Five studies reported the association between ARB usage and the risk of prostate cancer [15–17, 24, 25]. There was no significant relationship between ARB usage and the risk of prostate cancer according to the pool-analysis of all studies (RR1.09, 95% CI0.97–1.21). Subgroup analysis also suggested no significant connection between the use of ARB and the risk of prostate cancer according to the pooled-analysis of cohort studies (RR1.00, 95% CI0.83–1.20) and case-control studies (RR1.16, 95% CI0.98–1.38). However, heterogeneity among these studies was high (I^2^ = 83.7%) (Fig. 3).

### CCB and prostate cancer risk

**Fig. 4:**
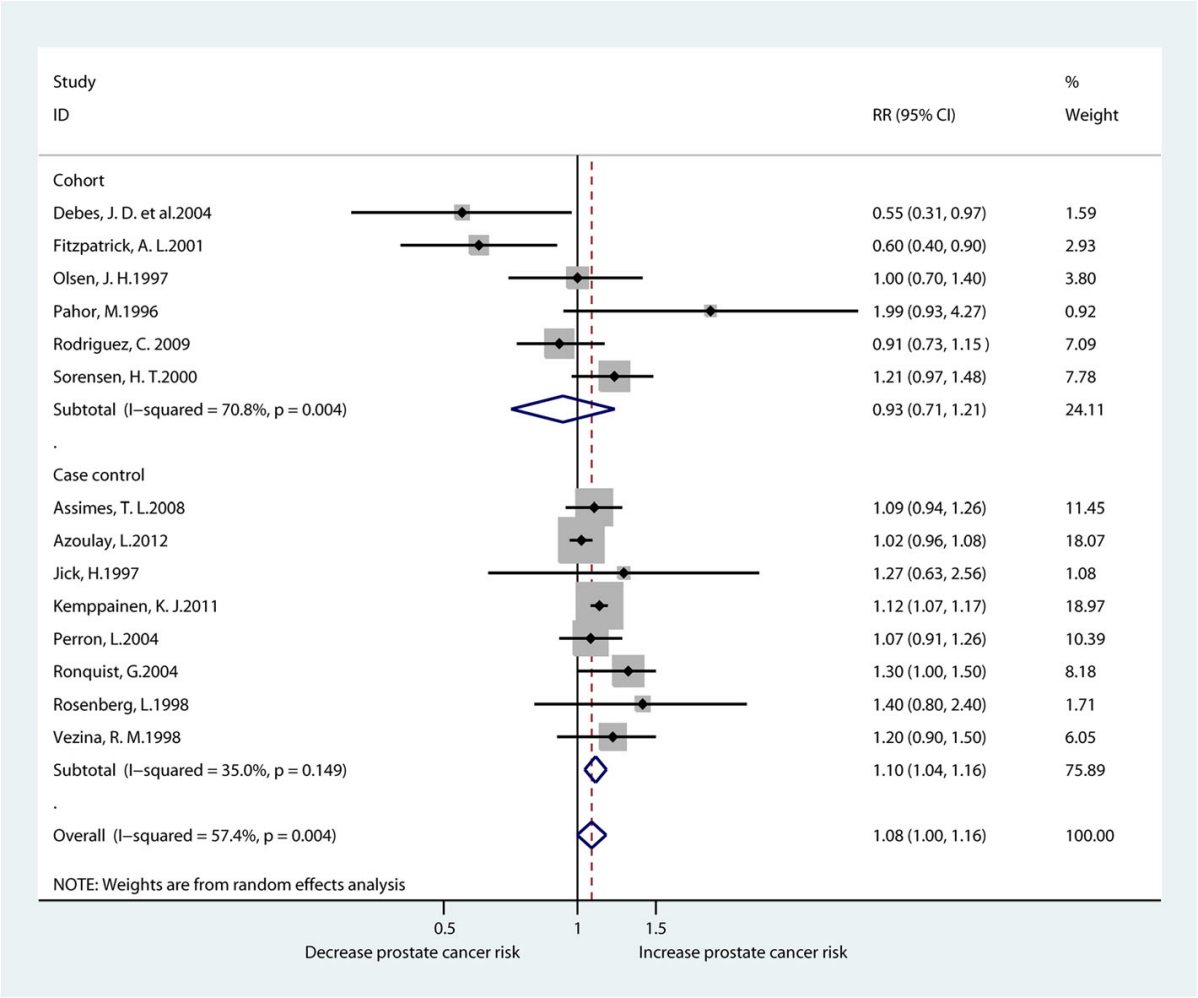
Forest plot for CCB use and prostate cancer risk (RR relative risk, CI confidence interval)

A total of 14 studies reported the connection between CCB usage and the risk of prostate cancer [10, 11, 15, 16, 19, 26, 31–35, 36–38]. There appeared to be a significant association between CCB usage and the risk of prostate cancer, according to the meta-analysis of all studies (RR1.08, 95% CI1.00–1.16). There was considerable heterogeneity existing among these studies (I^2^ = 57.4%). Subgroup analysis also indicated that without significant relationship between CCB usage and the risk of prostate cancer in terms of cohort studies (RR0.93, 95% CI0.71–1.21) but there was clear association between the use of CCB and the risk of prostate cancer according to the pool-analysis of case-control studies (RR1.10, 95% CI1.04–1.16) (Fig. 4).

### Beta-blockers and prostate cancer risk

**Fig. 5:**
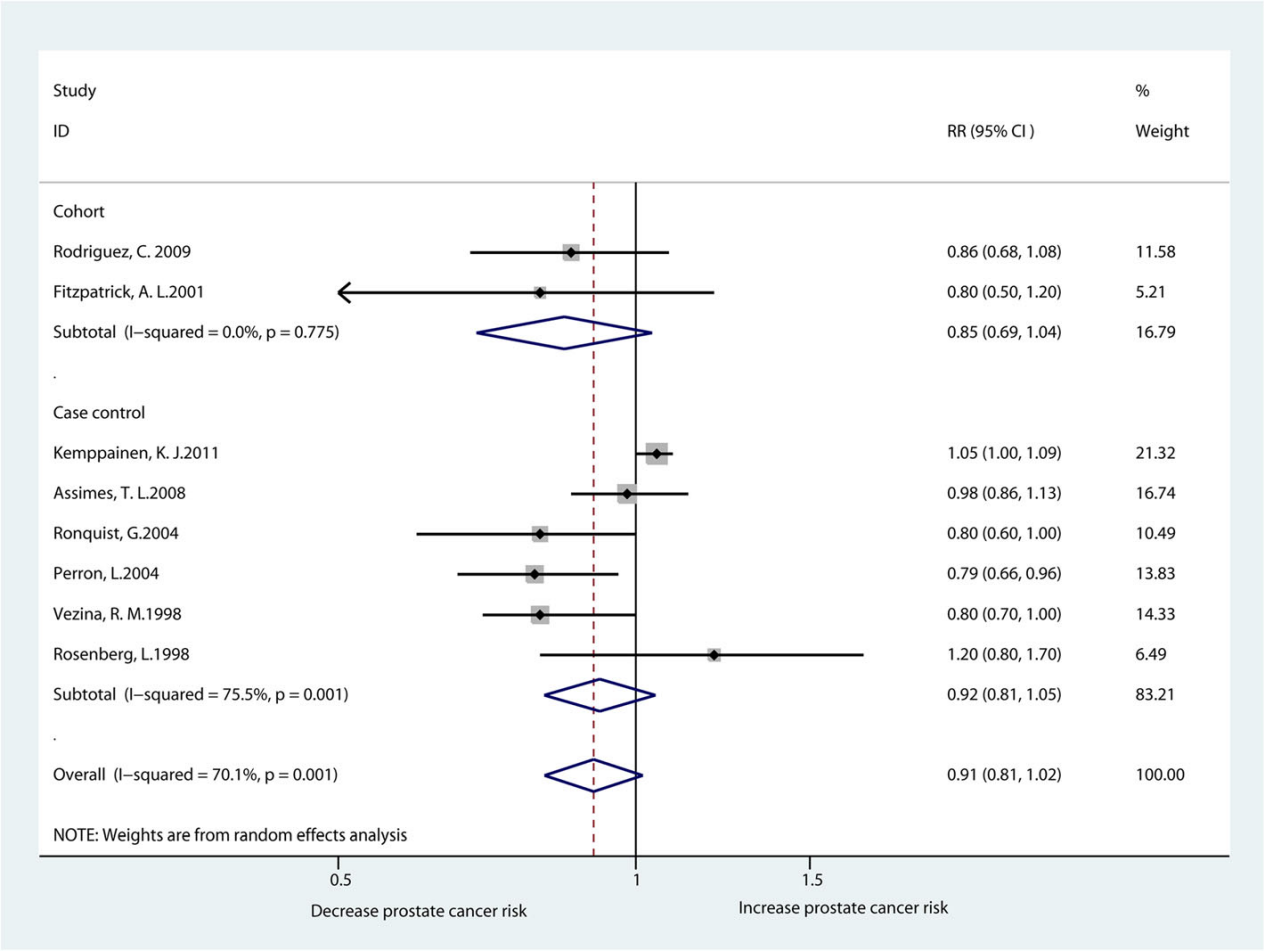
Forest plot for beta-blockers use and prostate cancer risk (RR relative risk, CI confidence interval)

There were 8 studies involving the subject of the relationship between beta-blocker usage and the risk of prostate cancer [15, 19, 26, 31, 34–37]. The meta-analysis of all studies suggested that there was no significant association between beta-blocker usage and the risk of prostate cancer (RR0.91, 95% CI0.81–1.02). We also did not find a significant connection between beta-blocker usage and the risk of prostate cancer, according to the pooled analysis of cohort (RR0.85, 95% CI0.69–1.04) or case-control studies (RR0.92, 95% CI0.81–1.05). There was significant heterogeneity existing among these studies (I^2^ = 70.1%) (Fig. 5).

### Diuretics or antiadrenergic drugs and prostate risk

**Fig. 6:**
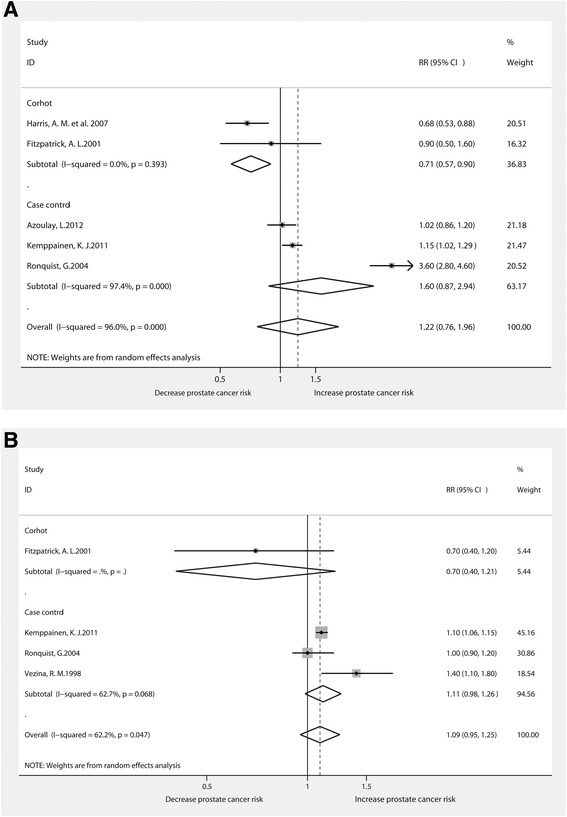
Forest plot for use of antiadrenergic agents or diuretic and prostate cancer risk: **a** antiadrenergic agents and **b** diuretic. RR relative risk, CI confidence interval

There were 5 and 4 studies involving the association between the use of diuretics [15, 31, 36, 35] and antiadrenergic drugs [15, 16, 28, 31, 35] and the risk of prostate cancer, respectively. We did not find significant association between the use of antiadrenergic drugs and the risk of prostate cancer (RR1.22, 95% CI0.76–1.96). The relationship between the use of diuretics and the risk of prostate cancer was demonstrated as not significant(RR1.09, 95% CI0.95–1.25). Significant associations between antiadrenergic drug usage and the risk of prostate cancer were found according to the cohort studies (RR0.71, 95% CI0.57–0.90) (Fig. 6).

## Discussion

To the best of our knowledge, this is first meta-analysis to investigate the relationship between the use of antihypertensive drugs and the risk of prostate cancer. Our main findings suggested that there was no significant association between the use of ACE inhibitors, ARB, beta-blockers, diuretics or antiadrenergic drugs and the risk of prostate cancer. However, CCB usage appeared to be associated with the risk of prostate cancer. Moreover, that considerable heterogeneity existed among the studies resulted in the reduction of evidence levels.

Prostate cancer is the most frequently diagnosed cancer diagnosed and the main causes of cancer death in older men in Western countries. Antihypertensive drugs such as ACE inhibitors and ARB are widely used for the management of hypertension and the prevention of cardiovascular disease events in high-risk persons [4, 40, 41]. Patients usually have long-term use of two or more different types of antihypertensive drugs to control their blood pressure [42]. The long-term ingestion of antihypertensive drugs can lead to adverse effects in patients, such as hypercholesterolemia, diabetes mellitus, chronic renal disease, and other cardiovascular diseases [43]. In addition, discussion of the connection between the use of antihypertensive drugs and the risk of cancer has consistently been a hot topic since the first relevant study was raised. We know that the older men are exposed to the condition of frequent use of antihypertensive drugs, and there is a high incidence of prostate cancer in this population [12, 44].

The topic of the relationship between the use of antihypertensive drugs and the risk of prostate cancer remain controversial, especially in the use of CCB and ACE inhibitors or ARB. The findings of a case-control study by Vezina, RM et al. suggested that there was no association between the use of CCB and the risk of prostate cancer in men younger than the age of 70 [36]. Debes, JD et al. found that there was an inverse association between prostate cancer and the use of CCB, and the result varied according to a family history of prostate cancer [14]. Loughlin KR reviewed relevant literature and thought that CCB had a protective effect on the development of prostate cancer on a basic science level, although the association in clinical practice has been controversial [45].

ACEI and ARB have been successfully used as potent antihypertensive drugs for a long period of time, and some literatures have suggested that these drugs could serve as new anticancer drugs [46]. The increased expression of AngII type 1 receptor (AT1R) mRNA was found in prostate cancer tissues compared with expression levels in the normal human prostate [47]. According to the data from basic science, ACE inhibitors or ARB might have a protective role in cancer [48]. A meta-analysis of randomized controlled trials (RCT) suggested that ARB are associated with an increased risk of new cancer diagnoses, while another meta-analysis of observational studies did not find significant associations between the use of ACE inhibitors or ARB and the risk of cancer, noting that the previous meta-analysis of RCT had a short duration of follow-up [9, 18]. In a case-control study, Hallas J et al. found significantly elevated OR for prostate cancer (OR 1.28, 95% CI 1.18, 1.39) in the patients using ACE inhibitors [17]. A cohort study by Rodriguez, C et al. indicated that there was no significant relationship between the use of ACE inhibitors and the risk of prostate cancer [26].

A meta-analysis suggested that the use of BB was associated with reduced specific mortality among patients with prostate cancer [49]. However, the relationship between BB usage and the risk of prostate cancer lacks consistent evidence. Perron, L et al. found that the long-term use of BB might prevent prostate cancer (OR 0.79, 95% CI 0.66, 0.96) [19]. However, Kemppainen, KJ et al. found that beta-blockers were associated with a marginally elevated risk of prostate cancer (OR 1.05, 95% CI 1.00,1.09) [15]. Fewer studies have paid attention to the relationship between the use of diuretics or antiadrenergic drugs and prostate risk compared with other types of antihypertensive drugs and their use also had different results [15, 20].

The present systematic review and meta-analysis provided a summary analysis of previous relevant studies which could yield a conclusion characterized by compromise. According to the pooled analyses of cohort studies, we generally did not find significant associations between the use of antihypertensive drugs and the risk of prostate cancer. Nevertheless, CCB usage might contribute to the higher risk of prostate cancer, based on findings from the case-control studies. Moreover, antiadrenergic drugs had a protective effect on the development of prostate cancer, according to the meta-analysis of two cohort studies. As we know, there was longer duration of follow-up in observational studies and we only reviewed these studies to explore whether the long-term use of antihypertensive drugs affected the incidence of prostate cancer in natural population. In the present review, most of the studies had a follow-up time of at least five years, and we had a larger sample to analyse than any previous studies. The main confounding factors, such as age, BMI, and race were adjusted for in most of the studies. Although we included studies with samples containing females, the relative risk was calculated under an adjustment for sex. Moreover, most of the studies were assessed with a high quality of methodology. As a result, our pooled analyses provided a conclusion that was more closer to the truth.

Our studies also had some limitations. First, our evidence grade was compromised by the considerable heterogeneity, which might be caused by various factors, including study design, race, follow-up time, social background. Second, small sample were analysed on the association between diuretics or antiadrenergic drugs and prostate risk, and the results should be cautiously considered. Third, we did not detect the publication bias, although it might exist among these studies. Finally, we did not conduct a dose-response meta-analysis due to the lack of relevant data in the included studies.

## Conclusions

We reviewed 21 observational studies and found that there was no significant association between use of antihypertensive drugs and the risk of prostate cancer. Nevertheless, CCB usage might contribute to the higher risk of prostate cancer based on findings from the case-control studies. Unfortunately, the high heterogeneity downgraded the evidence level and more well-designed studies with large samples are needed.

## Additional file

Additional file 1: Search strategy for relevant studies. (DOCX 12 kb)

## Abbreviations

ACEI: Angiotensin-converting enzyme inhibitors
ARB: Angiotensin II receptor blockers
CCB: Calcium-channel blockers
NOS: Newcastle-Ottawa Scale
RR: Relative risk
SMD: Standardized mean difference
