# Supplementary material for: Antihypertensive drugs use and the risk of prostate cancer: a meta-analysis of 21 observational studies
Source: BMC Urol. 2018 Mar 7;18:17. doi: 10.1186/s12894-018-0318-7 (PMC5842557; doi:10.1186/s12894-018-0318-7)
Supplement: Supplementary file 1 — Search strategy for relevant studies. (DOCX 12 kb) [file 12894_2018_318_MOESM1_ESM.docx]

**Additional file 1: Search strategy.**

**Pubmed**

“beta blockers” OR “angiotensin converting enzyme inhibitors” OR “angiotensin receptor blockers” OR “calcium channel blockers” OR “alpha blockers” OR “antihypertensive drugs” AND “prostate cancer”

**Embase**

“beta blockers” OR “angiotensin converting enzyme inhibitors” OR “angiotensin receptor blockers” OR “calcium channel blockers” OR “alpha blockers” OR “antihypertensive drugs” AND “prostate cancer”

**Science Citation Index**

“beta blockers” OR “angiotensin converting enzyme inhibitors” OR “angiotensin receptor blockers” OR “calcium channel blockers” OR “alpha blockers” OR “antihypertensive drugs” AND “prostate cancer”
